# Use of transoral outlet reduction endoscopy (TORE) in the management of resistant dumping syndrome

**DOI:** 10.1007/s00464-026-12620-z

**Published:** 2026-02-05

**Authors:** Arkeliana Tase, Mohamed Aly, Md Tanveer Adil, Aruna Munasinghe, Farhan Rashid, Periyathambi Jambulingam, Douglas Whitelaw, Vigyan Jain, Omer Al-Taan, Alan Askari

**Affiliations:** 1 Imperial College London, London, UK; 2 Luton and Dunstable University Hospital, Lewsley Road, Luton, Lu4 0DZ UK

**Keywords:** Dumping syndrome, Clinical pathways, TORE, Non-surgical methods

## Abstract

**Introduction:**

Dumping Syndrome (DS) and Reactive Hypoglycaemia (RH) are common occurrences post bariatric surgery, particularly post Roux-En-Y Gastric Bypass (RYGB). We present our initial results using Transoral Outlet Reduction Endoscopy (TORE) in the management of patients who failed to respond to dietary and medical treatment for DS.

**Methods:**

All patients identified to have symptoms consisting with DS were discussed in the complex bariatric MDT and assessed for suitability of TORE via an upper gastro-intestinal endoscopy to assess the length of the pouch, size of the gastro-jejunostomy and the presence of alternative pathologies.

**Results:**

Since the onset of our TORE services in January 2025, we identified 17 patients for treatment with TORE. The median age was 45yrs (IQR 36–55). Sixteen patients (94%) were women and all patients scored ≥ 7 on the Sigstad scoring questionnaire. Two patients had previously undergone conversion of gastric sleeve to a RYGB whilst all others had a primary RYGB. 2 patients were found to have unfavourable anatomy and was not safe to proceed with the procedure whilst one patient was followed up privately hence no data were available for review. The data showed a complete response to treatment at 2 years for 66% of patients. Four patients did not respond to treatment with TORE and are being considered for surgical intervention.

**Conclusions:**

TORE is an effective treatment for patients with DS not responsive to medical and dietary therapy. We believe it is an effective non-surgical treatment method prior to considering reversal of the original surgery (RYGB) with its associated weight regain. Further work is planned to assess its outcomes in larger groups of patients.

**Graphical Abstract:**

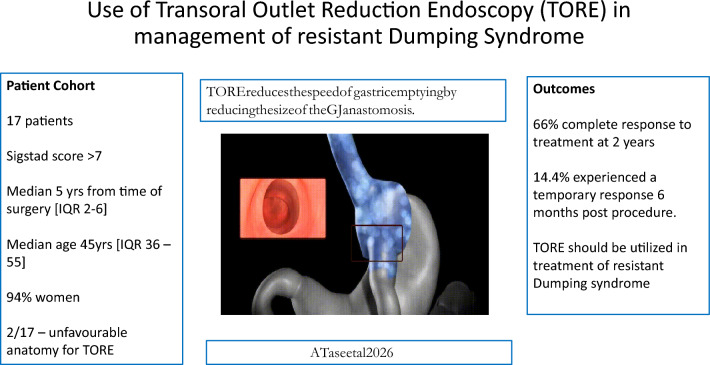

Dumping syndrome results from rapid emptying of gastric contents into the small intestine. It has been widely recognised as a consequence of gastric surgery and has in the recent years also been recognised as a complication of bariatric surgery [1–7].

Management of dumping syndrome is challenging. Numerous management methods have been reported in literature with dietary changes and medications such as acarbose and octreotide being widely used [7–11]. Variability in treatment methods is reported in literature with lack of standardised pathways in the management of these patients. Patients with DS and reactive hypoglycaemia can present with severe symptoms which have a significant effect on their quality of life [12].

In addition to medications and dietary changes, in the recent years TORE (Transoral Outlet Reduction Endoscopy) has been reported in the literature as a method of treating patients with DS and reactive hypoglycaemia (RG) [13, 14]. TORE was first developed in 2013 as an endoscopic procedure aiming to reduce the size of the gastro-jejunal (GJ) anastomosis in patients with weight regain post bariatric surgery [15]. The first study carried out in 2013 by Jirapinyo P et al [13] reported use of TORE for weight loss purposes. The authors reported technical success in reducing the anastomotic size to 6 mm with resulting weight loss up to 12 months post procedure. A more recent study showed an 8.8% total body weight loss at 5 yrs post TORE [14]. However, more recently, it has found use in the treatment of patients with DS by mechanically reducing the size of the gastro-jejunostomy. By doing so, it reduces the speed of pouch emptying [16, 17, 22–25]. 

The primary aim of this study was to review and present our outcomes and success rate of using TORE in the treatment of patients with DS resistant to medical and dietary treatments.

## Methods

Luton and Dunstable Hospital (Bedfordshire Hospitals NHS Foundation Trust) is a high-volume tertiary referral bariatric unit in the UK providing obesity care services encompassing a population of 11 million. We maintain a database of all patients referred to and operated on in out unit. We maintain a local Dendrite database which contributes to the UK-wide NBSR (National Bariatric Surgery Registry).

All our postoperative patients are followed up in outpatient clinic and asked questions in relation to symptoms of DS and RH. In patients with positive symptoms, a Sigstad [18] score is calculated. All symptomatic patients are then started on a management pathway and if clinically required, discussed in our Complex Bariatric MDT (held monthly) which includes a full spectrum of specialists, including obesity Clinical Nurse Specialists (CNS), obesity dieticians, clinical psychologists, obesity consultant physicians, consultant bariatric anaesthetists, and consultant bariatric surgeons.

In the last 2 years, we have gained expertise in using TORE as a new method of treatment and set up our services. Patients with symptoms of DS are initially seen by the dietician and obesity clinician to maximise dietary and medical treatment. Patients that continue to experience severe symptoms are reviewed by a bariatric surgeon to assess the possibility of TORE. Patients are counselled on the risks and benefits as well as the need for repeated procedures.

Currently, our follow-up period varies between 6 months to 2 years.

All patients with symptoms of DS resistant to other treatment are considered for TORE.

The following steps are followed at this stage.

### Pre-procedural evaluation

All patients undergo a pre-procedural diagnostic upper gastrointestinal endoscopy (OGD) carried out by a consultant bariatric surgeon, to assess the integrity of the gastro-jejunal anastomosis and exclude other potential causes of their symptoms. This is also an opportunity to assess the length and the general suitability of the gastric pouch for TORE. Commonly, a gastric pouch of at least 4 cm is required to make TORE feasible.

### TORE procedure

In selected candidates, a TORE procedure is carried out under General Anaesthetic, with the Apollo OverStitch Endoscopic Suturing System (Boston Scientific, Marlborough, MA, USA). Initially, a diagnostic procedure is undertaken, and an Argon probe is used to circumferentially etch the Gastro-jejunostomy (on the gastric pouch side) to generate fibrosis and circumferential narrowing of the Gastro-Jej anastomosis. Depending on the extent of narrowing required, an endoscopic suture is placed (OverStitch device – Boston Scientific) at 3 O’Clock and 9 O’Clock positions. This is repeated until the outlet is narrowed so that it only permits the OGD scope. The patient is then followed up and assessed for symptom resolution, based on which, further TORE procedure may be indicated.

These sutures effectively reduce the anastomotic diameter, aiming to slow gastric emptying and improve DS/RH symptoms [19]. Patients received an overnight hospital stay for observation in line with our protocol.

### Outcome measures

Our primary outcome measure was the rate of complete or partial resolution of symptoms of DS. For patients with partial symptom resolution, we aimed to assess the rate of improvement of symptoms. Patients were considered symptomatic post procedure if their Sisgstad scoring was ≥ 7.

### Sources of bias

We rely on the patient reporting of their symptoms for the diagnosis and assessment of symptom improvement. This brings with it some degree of reporting bias. To reduce further bias, the questionnaire and symptom recording were completed by the bariatric specialist nurses in their clinic without the presence of the treating surgeon.

### Ethical approval

The study was registered as a quality improvement project with Luton and Dunstable Educational department.

## Results

### Patient demographics

We started our TORE services in 2023. Since the onset, 17 patients were identified as candidates for the procedure and were consented for it. The median age was 45yrs (IQR 36—55). Sixteen patients (94%) were women and all patients scored ≥ 7 on the Sigstad scoring questionnaire. Two patients had previously undergone conversion of Sleeve gastrectomy to a RYGB (Roux-en-Y Gastric Bypass) whilst all others had a primary RYGB.

The procedure was successfully carried out in 15 patients. In 2 of the patients, the procedure was abandoned as the anatomy was considered unfavourable and not safe to proceed. One of the patients was followed up privately elsewhere and no follow-up data were available for reporting. 13 (93%) of the patients had one procedure with only one patient requiring a repeat procedure.

The median time between time of surgery and TORE procedure was 5 years [IQR 2–6 years].

### Proposed mechanism of action of TORE

TORE reduces the speed of gastric emptying by reducing the size of the GJ anastomosis. Existing studies from US and Germany support its use in patients with DS [15–17].

### Pre-TORE symptoms

Majority of the patients (n = 12) had symptoms 1–2 times daily. 1 patient had symptoms at least 5–6 times daily whilst the remaining patients presented with once weekly symptoms.

### Post TORE symptoms

Patients reported no symptoms of nausea or vomiting related to the TORE procedure itself. To date, the included patients have not experienced other complications such as ulcerations or stenosis of the gastro-jejunostomy. Three patients had no further symptoms post treatment and were discharged after a follow-up of 2 years post procedure. 4 patients reported only occasional symptoms (every 5–6 weeks), 2 had initial resolution but recurrence of symptoms at 6 and 8 months, 1 patient had improvement from daily to weekly and 4 patients had no improvement.

One patient underwent reversal of RYGB whilst the 4 who failed to respond to TORE are being considered for the next management step. Table 1 demonstrates the data in more detail.

**Table 1:** Outcomes of TORE in treatment of DS and RH

TORE	
Patients approved for treatment	17
Received treatment	15 (88%)
Technically not possible	2 (12%)
TORE success rate	66%
*Pre-operative symptoms*	
5–6 × daily + nocturnal	1 (7%)
1–2 daily	12 (86%)
Once weekly	1 (7%)
*Postoperative symptoms*	
Full resolution	3 (21.4%)
Every 5–6 weeks	4 (28.6%)
Temporary response	2 (14.4%)
Once weekly	1(7%)
No response	4 (28.6%)

## Discussion

We present here our results in using endoscopic treatment (TORE) in management of resistant DS.

Management of dumping syndrome is often difficult and challenging. It can be problematic post bariatric surgery and presents many challenges to the surgeon and patient alike. It is important to note that although DS and RH may present similarly and are considered part of the same process, differences are recognised in their pathology. DS is thought to occur due to rapid passage of food in the small intestine resulting in osmotic fluid shifts from the intravascular compartment to the intestine. In contrast, RH is thought to occur due to increased insulin sensitivity on a background of high weight loss. This leads to a hyperinsulinaemic episode leading to hypoglycaemia [26]. DS is reported to occur shortly after the surgery whilst RH typically occurs months to years postoperatively [11, 26]. A study from the Netherland [26] aimed to examine the relationship between DS and RH in bariatric patients [26]. They reported that DS is associated with GI and vasomotor symptoms whilst RH is defined by a hypoglycaemic event which starts 1–3 h after eating with hypoglycaemia-related symptoms (drowsiness, irritability and confusion) and adrenergic symptoms due to activation of the vagus nerve and sympathetic nervous system [10, 26]. 

Literature supports the use of small and frequent meals which include high protein intake and low glycaemic content [7, 20]. In those patients who fail to respond to conservative methods, ways to reduce postprandial glucose absorption leading to reduced insulin and GI hormone release are sought. Medications such as acarbose (alpha glucosidase inhibitors) have been tried with some effect in late dumping [7, 8]. Other medications used in treatment of both early and late dumping are somatostatin analogues (e.g. octreotide) which work by slowing gastric emptying and intestinal transit. Additionally, they reduce postprandial vasodilation and release of GI hormones [7–11]. 

Large-scale weight loss after bariatric surgery leads to increased insulin sensitivity which explains the benefits of weight loss surgery on diabetes. Additionally, surgeries such as RYGB have an endocrine effect on metabolic regulation by stimulating intestinal hormones that enhance insulin secretion [21]. 

Following initial medical therapy, our treatment methods are aimed at reducing the speed of gastric pouch emptying. We achieve this mechanically via the TORE procedure.

Studies from United States and Germany support the use of TORE as an adjunct to treating this group of patients with improvement in Sigstad scoring from 17± 6.1 to 2.6 ± 1.9 [16]. Brown et al [17] also demonstrated a 90% rate of symptom resolution although they only reported results at 3 months post procedure. Hakiza et al. concluded that TORE is effective in treating symptoms of DS [15]. Our experience showed that 66% of patients responded to TORE with an improvement in their symptoms.

## Conclusions

Our data have shown that TORE can be an effective tool in managing patients with resistant DS. Further work is required to study its outcomes in larger group of patients.

## Study limitations

This study is limited by presenting best practice by a single bariatric centre in UK. We are aware that variability in practices exists and clinical pathways are often adopted to the local protocols. We only recently started performing TORE in the last 2 years and the number of patients is low, however, the results are encouraging. A larger dataset is, however, required to fully assess its outcomes in the long term. Currently, the sample data were considered too small for a meaningful statistical analysis.
